# Disentangling the relationship between children’s motor ability, executive function and academic achievement

**DOI:** 10.1371/journal.pone.0182845

**Published:** 2017-08-17

**Authors:** Mirko Schmidt, Fabienne Egger, Valentin Benzing, Katja Jäger, Achim Conzelmann, Claudia M. Roebers, Caterina Pesce

**Affiliations:** 1 Institute of Sport Science, University of Bern, Bern, Switzerland; 2 Department of Psychology, University of Bern, Bern, Switzerland; 3 Department of Movement, Human and Health Sciences, University “Foro Italico” of Rome, Rome, Italy; Universita degli Studi di Verona, ITALY

## Abstract

Even though positive relations between children’s motor ability and their academic achievement are frequently reported, the underlying mechanisms are still unclear. Executive function has indeed been proposed, but hardly tested as a potential mediator. The aim of the present study was therefore to examine the mediating role of executive function in the relationship between motor ability and academic achievement, also investigating the individual contribution of specific motor abilities to the hypothesized mediated linkage to academic achievement. At intervals of ten weeks, 236 children aged between 10 and 12 years were tested in terms of their motor ability (t1: cardiovascular endurance, muscular strength, motor coordination), core executive functions (t2: updating, inhibition, shifting), and academic achievement (t3: mathematics, reading, spelling). Structural equation modelling revealed executive function to be a mediator in the relation between motor ability and academic achievement, represented by a significant indirect effect. In separate analyses, each of the three motor abilities were positively related to children’s academic achievement. However, only in the case of children’s motor coordination, the mediation by executive function accounted for a significance percentage of variance of academic achievement data. The results provide evidence in support of models that conceive executive function as a mechanism explaining the relationship that links children’s physical activity-related outcomes to academic achievement and strengthen the advocacy for quality physical activity not merely focused on health-related physical fitness outcomes, but also on motor skill development and learning.

## Introduction

The beneficial effects of regular physical activity (PA) on children’s physical [1] and mental health [2] are well known. Nonetheless, secular trends point on a decline in children’s PA levels [3], accompanied by reduced levels in physical fitness, such as cardiovascular endurance [4], and motor coordination [5]. The decrease in children’s PA levels and PA-related motor performances is not only alarming in terms of their health, but also in terms of their cognitive development, knowing that both motor and cognitive abilities are strongly interrelated with academic achievement [6, 7].

To capture the construct of human motor ability, over the last half century various systematizations have been proposed, all uniformly claiming its multidimensional nature [8–11]. According to the three-level model of Bös [11], there are five basic motor abilities: endurance, strength, speed, coordination and flexibility. Besides flexibility–which is seen as an anatomically determined personal performance prerequisite of the passive system of energy transfer–the basic motor abilities, can be described as being either more energetically-determined or more information-oriented, depending on their involvement of cognitive control processes. While, for example, endurance is localized on the one side of the continuum, being a more energetically-determined ability, motor coordination would be placed at the other end, being a more information-oriented ability [12]. The explosive expression of strength (i.e., muscle power), for example measured by the standing long jump, is situated in between, since it is contains both an energetically-determined and an information-oriented component. Based on the assumption of shared information processes in both motor and cognitive control [13], information-oriented motor abilities should be more strongly related to children’s cognitive abilities as the more energetically-determined ones. However, because research has lacked to include different motor abilities across the aforementioned continuum in one single study, the question whether specific motor abilities, such as aerobic endurance, muscular strength or motor coordination, contribute differentially to the development of cognitive abilities and academic achievement remains unanswered.

In the literature, interrelations between multifaceted physical activity outcomes and academic achievement have been established. Most studies have focused on the energetically-determined ability of endurance, frequently finding a positive relationship with academic achievement [14–19], which seems to be maintained over time [20]. Cross sectional [21] as well as longitudinal studies [22] found that being fit or improving aerobic fitness is associated with better academic achievement. Studies addressing muscular strength are less frequent and inconclusive, as some of them detected an association of strength or strength training with academic achievement [14, 23–26], whereas others did not [27, 28]. More consistent evidence emerges from recent studies which have shifted the focus toward motor coordination [16, 29], which is closely interrelated with cognitive processes [30], crucial for school performance [31, 32] and a significant predictor of academic achievement [33]. Thus, the summarized results support the notion that motor abilities are highly relevant for academic achievement.

Even if a sound theoretical framework elucidating the relationship between motor ability and academic achievement is still lacking, executive function (EF) seems to be a potential candidate [34]. The term “executive function” stands for a construct, consisting of several distinct, yet interrelated, cognitive processes being responsible for controlling and organizing goal-directed behaviour [35, 36]. EF can be divided in three core EFs [37, 38]. 1) Updating is the ability to keep relevant information in working memory and process this information further. 2) Inhibition is the ability to refrain from prepotent responses or to resist distractor interference. 3) Shifting is the ability to switch attention back and forth between multiple tasks, operations, rules, or mental sets. However, until now, the mediational role of EF is merely supported by piecemeal evidence on the linkage of EF to motor ability and to academic achievement.

EF seems to be differentially associated with specific motor abilities (endurance, strength and coordination). The bulk of cross-sectional research on endurance has found strong evidence for a positive association [39–43]. For example, Pontifex et al. [41] found that higher-fit preadolescent children, as compared to lower-fit counterparts, have a more efficient neural resource allocation that translates into better performance in a modified flanker task measuring EF. These results are consistent with those from the rare longitudinal studies [44, 45]. Chaddock and collegues [44] showed that one year after initial testing, more fit children still displayed better flanker performance than less fit children. Thus, evidence suggests that aerobic endurance not only is a correlate, but a long-term predictor of EF performance in children.

Instead, to our knowledge, the association of muscular strength to children’s EF has still not been tested. This is quite surprising, since studies with adolescents [26], younger and older adults [46, 47] have reported beneficial effects of strength training on EF. Although in the few developmental cross-sectional studies, muscular performance tests have been used [48, 49], they were always merged with other motor ability tests to obtain a global motor ability index, leaving the specific relationship between muscular strength and EF unrevealed.

Motor coordination has been strongly linked to EF, in both typically developing children [29] and in children affected by developmental coordination disorder [50, 51]. Studies of this line of research have mainly investigated fine motor coordination with lesser studies focussing on the association of gross motor coordination with EF in children and adolescents [52–54]. However, using the Körperkoordinationstest für Kinder to measure gross motor coordination and the planning scale of the Cognitive Assessment System to measure executive functions in preadolescent children, Luz and colleagues [52] could show that children with higher levels of gross motor coordination displayed better cognitive performance then their lower-level counterparts.

In sum, while studies on the strength-EF relationships are still inconclusive, both the more energetically-determined ability of aerobic endurance and the more information-oriented ability of motor coordination seem correlated with EF performance. However, more research is still needed to understand whether peculiarities exist in their individual relation to EF.

High levels in EF, in turn, predict school readiness in young children [55] and explain a substantial amount of variance in elementary school children’s academic achievement [56]. The basic assumption behind the relationship between EF and academic achievement is that updating, inhibition and shifting must be coordinated to execute and monitor the sequences of actions necessary to successfully perform academic tasks as solving a new complex mathematical problem or comprehending a complex phrase [57, 58].

In explaining the potential mechanisms underpinning the relationship between motor ability and academic achievement, arguments ranging from neurophysiological to psychological explanations can be found in the literature. Research on aerobic endurance commonly refers to the *cardiovascular fitness hypothesis* [59], assuming that regular PA leads to functional and structural changes in those brain regions, which are especially relevant for learning and thus academic achievement [60]. At a functional level, studies including measures of event-related brain potentials (ERPs) have supported this claim, showing that behavioural indices of cognitive performance of higher-fit children were paralleled by a modulation of ERP components reflecting a larger and more efficient allocation of attentional resources during stimulus engagement [61, 62].

A less established psychological mechanism that is attracting increasing attention is related to the cognitive demands inherent in coordinatively challenging and goal-directed physical activities. The assumption of the *cognitive stimulation hypothesis* is that coordinatively demanding and non-automated movement and sports actions engage the same brain regions that are used to control higher-order cognitive processes [63–65]. Thereby, it is quite conceivable that those children, who get aerobically fit through, for example, regular soccer training, not only train their endurance. They also engage in regular motor skill learning, which is mostly both cognitively and coordinatively challenging and has been recently proposed a means to enhance cognitive abilities [66]. Thus, training sport-specific skills not only fosters cardiovascular fitness, but also motor coordination, which in turn challenges EF. This postulated mechanism is supported by results from basic research claiming particularly complex motor tasks to be appropriate to investigate the link between action and cognition [67], but also by few applied research studies in children and adolescents comparing designed physical activities with higher cognitive and/or coordinative demands to a more automated PA [68–71].

Despite the aforementioned interrelatedness of children’s motor ability, EF and academic achievement, to date only two cross-sectional studies [18, 53] have tested the hypothesized mediational role of EF [34] and one longitudinal study [55] has indirectly supported it. Interestingly, deriving from different disciplines (developmental psychology vs. sport science), they focus on two distinct PA outcomes: motor coordination and physical fitness, respectively. Rigoli and colleagues [53] revealed working memory of adolescents being a mediator in the relationship between motor coordination and academic achievement. Interestingly, of the three tested sub-components of motor coordination ability–manual dexterity, balance, and aiming/catching–only the latter resulted to have an indirect effect on school achievement mediated via children’s working memory. Van der Niet et al. [18] showed that physical fitness was significantly associated with EF as well as with school performance. Importantly, the direct effect from fitness to academic achievement resulted to vanish after introducing the mediating EF variable–a combined index of shifting and problem solving–in the model, which indicates a full mediation. A similar role of EF also emerged in a prospective study of the relationship between fine motor coordination and later academic achievement [55].

The aforementioned studies have either addressed multiple facets of motor coordination, but including only working memory rather than all three core EFs in their model [53], or incorporated only fine motor skills [55] or physical fitness measures [18] to evaluate their linkage to different sets of core and higher-order EFs. Thus, a comprehensive and differentiated view on the mediated path leading from children’s motor ability to academic achievement is still lacking. Therefore, the present study aimed to examine whether the relationship between motor ability and children’s academic achievement was mediated by their EF, including all three core EFs in consideration of their unity and diversity [38]. Since testing mediation in cross-sectional data can produce biased and potentially misleading estimates of the mediational process [72], the analysis was conducted on longitudinal data. To reveal whether specific motor abilities predict EF and academic achievement differentially, three separate mediation analyses were performed for endurance, strength and motor coordination.

## Materials and methods

### Design

Institutional Review Board of the Faculty of Human Sciences at the University of Bern. Approval number: 2013-1-292892.

At intervals of ten weeks, a sample of preadolescent children was tested in terms of their motor ability (t1), EF (t2), and academic achievement (t3). To ensure that the sample was representative and to control for potential confounding variables, during t1, information about PA level, pubertal and socioeconomic status was collected using questionnaires, heights and weights (for calculating the body mass index, BMI) were measured.

### Participants

A total of 236 children ranging from 10 to 12 years of age (*M* = 11.30 years, *SD* = 0.62; 52.5% girls) were included in the analyses (Table 1). Out of the original data-set (*N* = 237), one case had to be excluded, since according to the critical values for chi-squared (χ^2^) distributions, it was identified as a probable multivariate outlier, having a Mahalanobis distance greater than 27.877. Mahalanobis distance values were calculated as χ^2^ at *p* < .001 with 9 degrees of freedom [73]. The percentage of children with incomplete values was 3.4% at t1, 4.6% at t2, and 12.1% at t3. Data loss was due to sick leave, non-participation in the motor ability tests because of injury, or incomplete questionnaires. However, since Little’s MCAR test was not significant (χ^2^ (39) = 38.44, *p* = .495), missing values can be interpreted as missing completely at random. Thus, missing values were treated by applying AMOS’s regression imputation.

**Table 1:** Descriptive statistics and mean differences between all variables by gender.

	Boys (*n* = 112)	Girls (*n* = 124)	Total (*n* = 236)	*t* (234)	*p*	*d*
	*M (SD)*	*M (SD)*	*M (SD)*			
**Sample characteristics**						
Age (years)	**11.31 (0.63)**	**11.30 (0.60)**	**11.30 (0.62)**	**0.25**	**.807**	**0.016**
Physical activity level (mean)	**3.32 (1.86)**	**2.86 (1.38)**	**3.08 (1.64)**	**2.14**	**.034**	**0.281**
Pubertal status (sum score)	**4.11 (1.07)**	**5.43 (1.69)**	**4.82 (1.57)**	**7.21**	**< .0005**	**0.933**
Socioeconomic status (sum score)	**6.70 (1.55)**	**6.31 (1.67)**	**6.49 (1.62)**	**1.84**	**.068**	**0.242**
Body mass index (kg/m^2^)	**17.99 (2.94)**	**17.77 (2.52)**	**17.87 (2.72)**	**0.62**	**.538**	**0.080**
**Model variables**						
Endurance (mL∙kg^-1^∙min^-1^)	**50.62 (6.07)**	**46.38 (5.26)**	**48.39 (6.03)**	**5.75**	**< .0005**	**0.747**
Strength (cm)	**160.41 (18.24)**	**157.24 (21.87)**	**158.75 (20.25)**	**1.22**	**.225**	**0.157**
Coordination (correct jumps)	**37.44 (5.72)**	**36.07 (6.21)**	**36.72 (6.01)**	**1.76**	**.079**	**0.229**
Updating (correct answers)	**11.50 (2.95)**	**11.82 (2.59)**	**11.67 (2.76)**	**0.90**	**.369**	**0.115**
Inhibition (ms)	**62.28 (53.66)**	**58.19 (52.65)**	**60.13 (53.06)**	**0.59**	**.555**	**0.077**
Shifting (ms)	**418.85 (145.15)**	**427.10 (155.86)**	**423.19 (154.77)**	**0.41**	**.683**	**0.055**
Math (correct answers)	**16.38 (5.80)**	**14.52 (6.43)**	**15.40 (6.20)**	**2.33**	**.021**	**0.304**
Reading (reading quotient)	**103.38 (16.25)**	**105.99 (15.97)**	**104.76 (16.12)**	**1.25**	**.215**	**0.162**
Spelling (correct answers)	**35.30 (7.31)**	**35.65 (6.21)**	**35.48 (6.74)**	**0.40**	**.690**	**0.052**

Both the principals of the schools and the parents of the children signed an informed consent form approved by the Institutional Review Board of the Faculty of Human Sciences at the University of Bern (approval # 2013-1-292892) prior to participating in the study. All the children were asked before the first data collection session whether they wanted to participate and informed that they could discontinue at any time during the study. All data were treated confidentially.

### Measures

#### Motor ability

Motor ability was tested using three standardized tests to obtain measures of a more energetically-determined (aerobic endurance), a more information-oriented (motor coordination) and an intermediate motor ability dimension (muscular strength). To avoid the metabolic, muscular and motor coordination demands being coupled with large differences in movement skills involved, three tests were chosen, which have communalities in fundamental movement skills type (i.e. locomotor skills, not object control or stability skills; e.g. [74]). Furthermore, the “intermediate dimension” of muscular strength was conceptualized as a motor ability containing both an energetically-determined *and* an information-oriented component.

*Endurance* was assessed using the Multistage 20-Meter Shuttle Run test [75]. Participants had to run back and forth along a 20 m course and touch the 20 m line with their foot when a sound signal was emitted from a pre-recorded tape. The frequency of the sound signals was increased every minute, by 0.5 km/h, starting with a speed of 8.5 km/h. The test ended when participants failed twice in succession to reach the line before the signal sounded. The test score is the time achieved in seconds. Maximal oxygen uptake (VO_2_max; mL ∙ kg^-1^ ∙ min^-1^) was estimated from the number of the last stage reached as: 31.025 + (3.238 ∙ velocity)–(3.248 ∙ age) + (0.1536 ∙ age ∙ velocity). Evidence for the acceptable reliability and validity of the 20 m shuttle run test has been proven, with test-retest reliability coefficients ranging from *r* = 0.78 to *r* = 0.93 [76].

*Strength* was assessed using the standing long jump [77]. The standing long jump is a field test to measures the explosive strength of the lower extremities. Participants have to stand behind a starting line and jump with both feet as far as possible. The test score (best of two trials) is the distance in centimetres, measured form the starting line to the point where the back of the heel landed on the floor. Evidence for the acceptable reliability and validity of the test in 6- to 12-year-olds has been proven [78]: Test-retest reliability was *ICC* = .94. Concerning validity, the standing long jump showed a strong association with the One Repetition Maximum Leg Extension Test [79] with an adjusted coefficients of determination of *R*^2^ = .70 after controlling for weight, height, sex, and age.

*Coordination* was measured by jumping sideways, a subtest of the Körperkoordinationstest für Kinder (KTK; [80]). In a field of 60 × 100 cm, framed by side lines and divided by a centre line, participants have to perform as fast as possible consecutive jumps from side to side. Jumps in which the participant steps either on the centre or the side lines are not counted. The feet have to be kept together. The test score is the sum over all correct jumps over two trials within 15 seconds. With a test-retest reliability of *r* = .95 and with 91% of children with brain damage being differentiated from normal children, acceptable reliability and validity seems to be given [80].

#### Executive function

EF was measured in two computer-based tasks using E-Prime Software (Psychology Software Tools, Pittsburgh, PA). Each task took about 10 minutes to complete and the order of the two tasks was counterbalanced between participants.

*Updating* was assessed by means of a non-spatial n-back task (adapted from a spatial n-back task [81]). Several pictures of fruit were presented one after another on the screen. Children were instructed to press the right button in front of them when the fruit on the screen was similar to the second to last fruit presented (target) and the left button in all other cases (non-targets). They completed two practice blocks containing 10 trials each before they started with the two test blocks. The n-back task consisted of two test blocks containing 24 trials each, with one third of all trials being targets. The total number of correct answers was used as the dependent measure.

*Inhibition* was measured by means of a child-adapted Eriksen flanker task [82] consisting of a block with 20 congruent trials (“pure” block) and a block with 20 congruent and 20 incongruent trials (“standard” block) in a randomized order [83]. This fish flanker task is considered as the child version of the Attention Network Test [84], which has widely been used in developmental research [13, 85, 86] also with specific regard to exercise and cognition studies [68, 83, 87]. In order to check whether the children understood the fish flanker task, they completed five practice trials before each block and were lead into a feedback loop with additional practice trials if performance was below 60% accuracy. The conflict score between trials with the highest rate of distraction (incongruent trials standard block) and trials with the lowest rate of distraction (congruent trials pure block) was calculated as the dependent measure for inhibition [85].

*Shifting* was assessed by an additional block (“mixed” block) included in the flanker task [68, 83]. In this block, again, 20 congruent and 20 incongruent trials were shown and an additional rule was introduced–cued by a different color of the trials. Children had to adapt their response depending on the color of the trials, requiring a switch between the two rules whenever the color of the trials changed. Global switch costs were calculated as the dependent variable [88]. Since trials in the mixed block not only required the child to shift between different tasks, but also contained inhibitory demands, the difference between the mixed and the standard block was calculated to control for the inhibition component.

#### Academic achievement

Academic achievement (math, language) was assessed using three standardized academic achievement tests.

*Math performance* was measured using the three subscales (arithmetic, geometry, and solving written math problems) of the German math test for 5^th^ graders (Deutscher Mathematiktest für fünfte Klassen DEMAT 5+; [89]).

*Reading* was assessed using the Salzburger Lese-Screening für die Klassenstufen 5–8 (SLS 5–8; [90]).

*Spelling* was assessed using the Hamburger Schreib-Probe 1–10 (HSP 1–10; [91]).

#### Background variables

The Physical Activity Questionnaire for Children (PAQ-C; [92]) was used to measure *general levels of PA*. The PAQ-C is a 7-day self-administered recall measure that provides a summary PA score derived from nine items. The response format varies by item, but each is scored on a 5-point scale, a sample item being: “In the last 7 days, on how many evenings did you do sports, dance, or play games in which you were very active?” Response options range from: “None” (1 point) to “6 or 7 times last week” (5 points). Evidence for acceptable reliability and validity of the questionnaire in 8- to 16-year-olds has been provided by Crocker et al. [92].

The German version [93] of the Pubertal Development Scale (PDS; [94]) was used to assess *pubertal status*. For each gender, three questions are used to determine the pubertal status, a sample question for boys being: “Have you noticed a deepening of your voice?” Response options were: not yet started (1 point); barely started (2 points); definitely started (3 points); seems complete (4 points). The puberty index (ranging from 3 to 12) was calculated from the sum of the three items. Evidence for acceptable reliability and validity of the German version used in 9- to 13-year-olds has been provided by Watzlawick [93].

The Family Affluence Scale II (FAS II; [95]) was used to assess the *socioeconomic status*. The scale consists of four questions asking children about things they are likely to know about in their family (number of family-owned cars, computers, number of family holidays in the past year, and having an own bedroom at home). A sample item is: “Does your family own a car, van or truck?” Response options are: no (0 points); yes, one (1 point); yes, two or more (2 points). The response format varies by item. The prosperity index (ranging from 0 to 9) was calculated from the sum of the four items. Evidence for acceptable reliability and validity has been provided by Boudreau and Poulin [95].

The *BMI* was calculated as the body weight (in kg) divided by the square of the height (in m).

### Procedure

After receiving the principals’ permission, 16 fifth-grade teachers were contacted, who agreed to participate in the study. To investigate the longitudinal relationship between children’s motor ability, EF and academic achievement, three waves of data (4 weeks each) were collected at intervals of 10 weeks. At t1, after filling out the questionnaires in the classroom, three trained research assistants carried out the three motor ability tests in the gym of the respective schools. All tests took place during physical education classes in the morning. First, children were randomly assigned either to the jumping sideways or to the standing long jump test. Once all children had completed these two tests, the shuttle run test was performed. The same procedure was performed a second time during the four weeks of t1 to obtain two measures of the respective motor ability. At t2, cognitive testing was set between 10.00 a.m. and 12.00 p.m. for all participants and took place in a quiet room in small groups of four children. First, one investigator gave some general instructions. All the cognitive tasks were then completed on a computer and children received the instructions both in writing on the screen and simultaneously over headphones, which at the same time served as sound absorbers. At t3, three standardized academic achievement tests were conducted in random order. These tests were performed between 10.00 a.m. and 12.00 p.m. and took place in the classroom.

### Statistical analysis

**Fig 1:**
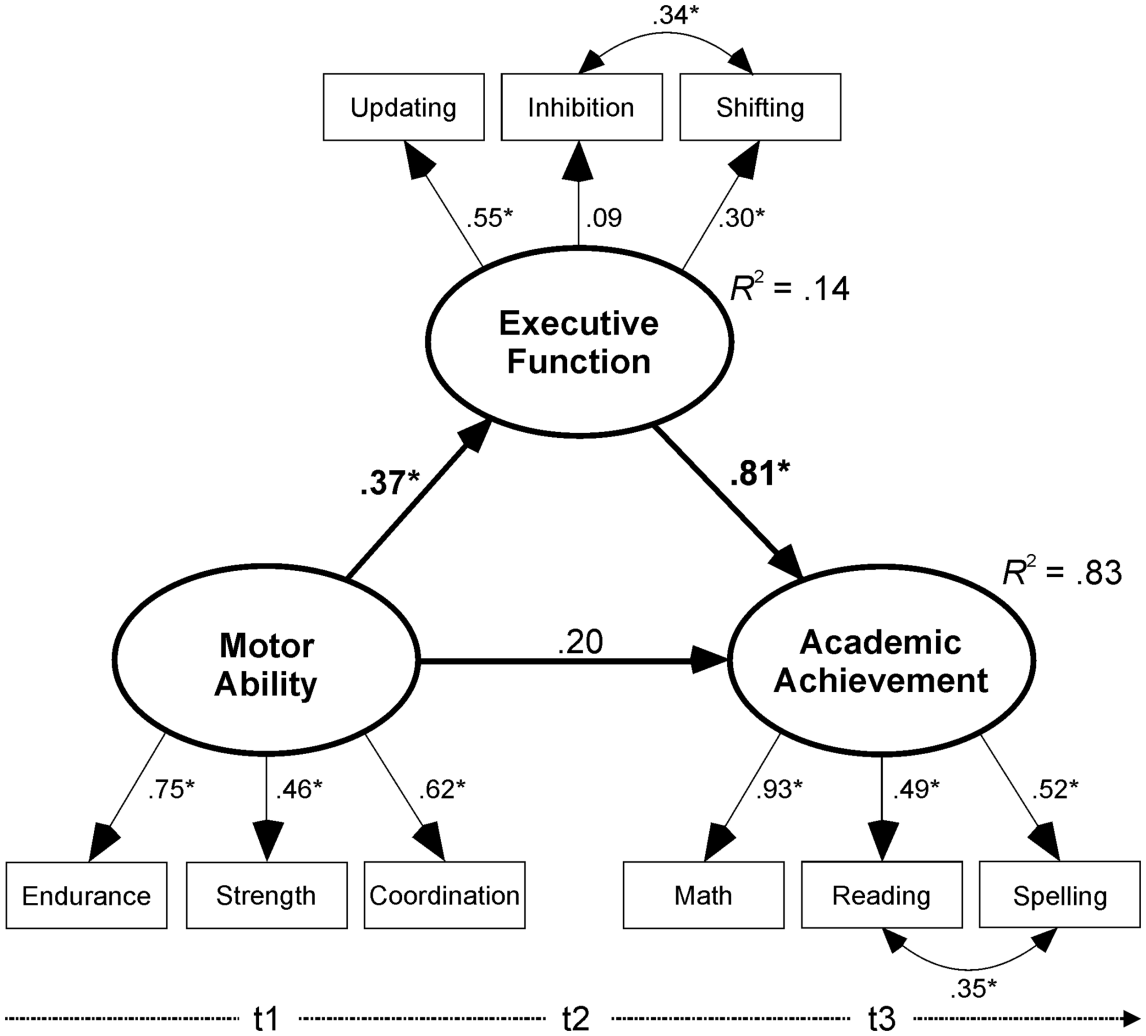
Mediation model, with motor ability as the predictor, executive function as mediator, and academic achievement as outcome variable.

Confirmatory factor analysis and structural equation modelling were realized using AMOS 24 software. As a first step, a confirmatory factor analysis was performed to test whether the latent variables (motor ability, EF, and academic achievement) were described adequately by the observed measures. In a second step, a structural equation model was designed with a direct path from motor ability to academic achievement and a direct path from motor ability to EF and from EF to academic achievement (Fig 1). To control for age and socioeconomic status [96–99], in all models direct paths were drawn from these two manifest variables to all latent variables. To test the hypothesized mediating role of EF, bias-corrected bootstrap analyses (95% BC confidence level; [100]) were performed, to reveal the indirect effects as significantly different from zero [101]. Finally, to test the specific contribution of the three different motor abilities, the aforementioned mediation analyses were performed in three separate models with endurance, strength, or coordination as a latent predictor variable. To assess model-data fit, standard indices were calculated and compared with the criteria for acceptable fit [102]: the χ^2^ statistic; comparative fit index (CFI, with values equal to .95 or better); the root mean square error of approximation (RMSEA, which should be .08 or less); and the standardized root mean square residual (SRMR, with .10 or less for a good model fit). To facilitate the comparison with other studies, all path coefficients are presented as standardized estimates. A significance level of .05 was set for all tests. When effect size was calculated, it was interpreted by means of Cohen’s [103] definition of small, medium, and large effects (Cohen’s *d* = .20, .50, .80, respectively).

All reported path coefficients (bold when significant, *p* < .05) are standardized estimates. *R*^2^ = coefficient of determination, indicating the proportion of the variance in the dependent variable that is explained by the independent variable(s).

## Results

### Confirmatory factor analysis

The model with all three latent variables linked by covariances provided good fit with the data (χ^2^(22) = 26.90, *p* = .215, CFI = .987, RMSEA = .031, SRMR = .055). Except inhibition, all manifest variables loaded significantly on the respective latent variable and small to large amounts of the item variance were explained (*R*^2^ ranged from .09 to .92). Based on the good model fit, inhibition was not excluded from the model despite its non-significant factor loading. The covariances between the latent variables were all significant at *p* < .05.

### Mediation analysis

To test the mediation hypothesis, the covariances in the aforementioned model were replaced by directional paths from motor ability to academic achievement, from motor ability to EF, and from EF to academic achievement. Since multi-group analyses conducted for all models revealed no significant effect of gender on models (all CFI differences < .01) or on single path coefficients (all critical ratios < 0.9), only the analyses on the entire sample are reported here. Age (β = .20, *p* = .017) and socioeconomic status (β = .22, *p* = .009) were identified as significant predictors of children’s motor ability, but not of EF or academic achievement (*p*s > .318). The direct path from motor ability to academic achievement, without EF as mediator, was significant (β = .47, *p* = .001, *R*^2^ = .23). This path coefficient decreased to a non-significant level when EF was included as a mediator (β = .20, *p* = .435). The paths from motor ability to EF (β = .37, *p* = .021) as well as from EF to academic achievement (β = .81, *p* = .002) were significant and the model fit was very good (see Table 2). Most importantly, the indirect effect by EF proved to be significant (β = .30, *p* = .020), indicating its mediating role from motor ability to academic achievement.

**Table 2:** Goodness of fit statistics for the estimated models compared with recommendations for model evaluation by Schermelleh-Engel et al. [102].

Model	χ^2^	*p* (df)	χ^2^/df	CFI	RMSEA	SRMR
A.S.		≥ .05	≤ 3	≥ .95	≤ .08	≤ .10
Motor ability model	33.97	.469 (34)	0.99	1.00	< .0005	.049
Endurance model	25.48	.436 (25)	1.02	.999	.009	.046
Strength model	14.96	.942 (25)	0.60	1.00	< .0005	.034
Coordination model	18.96	.799 (25)	0.76	1.00	< .0005	.039

*A*.*S*. = Accepted Standard for Good Fit; *CFI* = Comparative Fit Index; *RMSEA* = Root Mean Square Error of Approximation, *SRMR* = Standardized Root Mean Square Residual. In all models, age and socioeconomic status are controlled.

**Fig 2:**
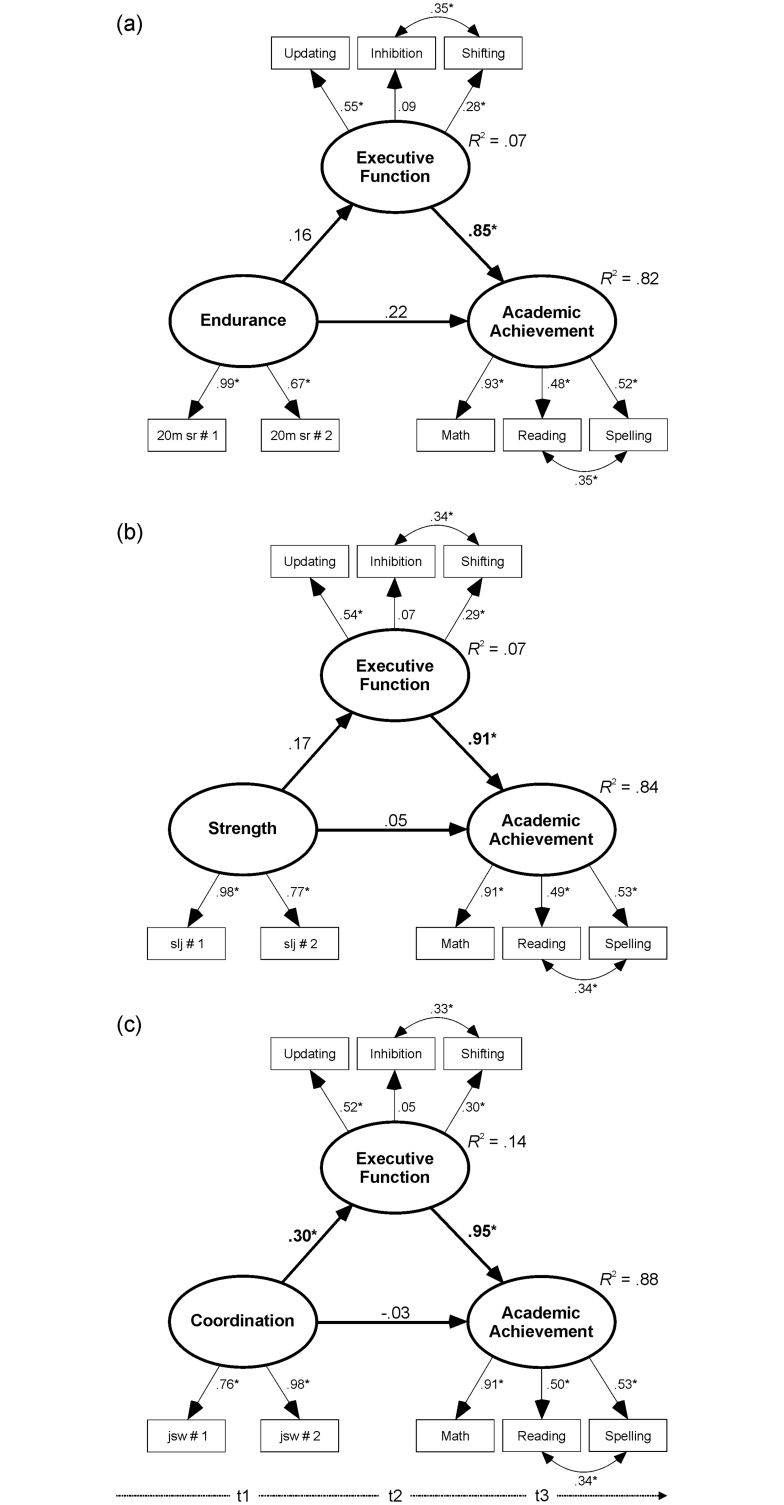
Mediation models of the three specific motor abilities of a) endurance, b) strength and c) coordination.

Three separate models were set up to test the unique contribution of each single motor ability. To be able to perform this mediation analysis by means of a latent (and not a manifest) variable approach, the two individual test scores of endurance, strength and coordination, were used to form the corresponding latent variables. Without considering EF as a mediator, in all three models, the direct paths from each single motor ability to academic achievement were significant (endurance: β = .33, *p* < .0005, *R*^2^ = .13; strength: β = .18, *p* = .001, *R*^2^ = .06; coordination: β = .23, *p* = .001; *R*^2^ = .09). However, as can be seen in Fig 2, after including EF into the models, only in the coordination model, the direct path to EF was significant (β = .30, *p* = .018), resulting in a significant indirect effect of coordination on academic achievement (β = .29, *p* = .018). In contrast, in the endurance and strength models, both the direct effect to EF (endurance: β = .16, *p* = .205, strength: β = .17, *p* = .092) and the indirect effect on academic achievement were not significant (endurance: β = .13, *p* = .214, strength: β = .15, *p* = .091).

All reported path coefficients (bold when significant, *p* < .05) are standardized estimates. *R*^2^ = coefficient of determination, indicating the proportion of the variance in the dependent variable that is explained by the independent variable(s).

## Discussion

The aims of the present longitudinal study were to investigate (i) whether EF plays a mediating role in the relationship between motor ability and academic achievement in preadolescent children and (ii) whether specific motor abilities contribute differentially to the hypothesized mediated linkage. The results showed that (i) the relationship between motor ability and academic achievement was indirectly mediated by EF and that (ii) among the three different motor abilities–all positively related to children’s academic achievement–only children’s motor coordination ability predicted their academic achievement fully mediated through their EF performance.

Going beyond the mere accumulation of empirical evidence on the interrelation of motor ability and academic achievement, the present study aimed to contribute developing a sound theoretical framework in search for possible mediating mechanisms *explaining* the relationship between these variables. The present longitudinal approach [72] chosen to test the mediational role of EF in the linkage between motor ability and academic achievement [34] confirms and further strengthens the mediated path found in cross-sectional studies [18, 53]. In the present study and those by Rigoli et al. [53] and van der Niet et al. [18], there are strong similarities in path coefficients despite of considerable differences in age groups, procedure and instruments used to operationalize the underlying constructs. This underscores the interrelated role of motor ability and EF as predictors of academic performance in children and adolescents in any way measured.

The results of the present study support the unique relationship between the developmental trajectories of motor coordination and higher-level cognition [6, 50, 62] and the potential that this relationship has to translate into successful academic achievement. Indeed, EF is not only predictive of successful achievements in school and life [37], but also especially sensitive to PA and therefore associated to PA outcomes [104, 105], including qualitative changes in motor coordination competence. In addition, deficits in motor coordination (e.g., developmental coordination disorder) seem associated with poor literacy and numeracy skills [106] and psychosocial difficulties [30, 50].

The positive association between children’s motor ability, conceptualized as a latent variable, and their academic achievement is in line with the few studies also adopting a latent variable approach [18, 53]. Interestingly, in our study, the latent variable covering the entire range from more energetically-determined to more information-oriented motor abilities explained almost twice as much variance of academic achievement (*R*^2^ = .23) as any single ability (*R*^2^ = .06-.13). This points on the importance of operationalizing children’s motor ability according to a holistic view on the structure of human motor ability that incorporates multiple motor abilities to best represent its multi-dimensional nature [8–11].

The novelty of the present study is certainly the individual and joint consideration of three different motor abilities to better understand their relation to higher-order cognition and academic achievement, revealing the unique contribution of motor coordination (Fig 2). Although each single motor ability significantly predicted academic performance, only the motor coordination model revealed a full mediation through EF performance. This full mediation is mainly due to the strong correlation between motor coordination and EF, which leads to a double-size effect (*R*^2^ = .14) as compared to the other two motor abilities (*R*^2^ = .07). This special role of motor coordination is in line with the outcomes of rare exercise and cognition studies including different motor abilities in their analyses [27, 49, 107].

The results from the three separate mediation models applied to the linkage between different motor abilities, EF and academic achievement also shed light into the underlying mechanisms. Besides well-grounded neurophysiological explanations for the relationship between motor ability and academic achievement [60, 108], research has shifted the focus from exercise-related metabolic, neurophysiological and neurotrophic mechanisms to more psychologically informed constructs, potentially serving as mediators [64, 109, 110]. The most frequent discussed mediator, as mechanism of action, is clearly EF [34]. Why exactly EF can be considered a viable candidate to mediate the relation between motor ability and academic achievement has been explained differently. Some of the shared variance between motor coordination and EF might be attributable to the speed-accuracy trade-off, which is inherent in most common coordination as well as most EF tasks [13]. However, motor coordination may also be associated with EF because both are required and therefore trained by regular physical activity and cognitively challenging sports [65, 110]. To succeed in most sports, individuals have to adapt their goal-directed behaviour to a constantly changing environment. In more detail, especially those physical activities, which consist in high amounts of novelty, diversity and complexity, are thought to stimulate improvements in EF [110, 111].

Specifically designed physical activities, including physical and cognitive demands, have been shown to be more effective in promoting EF compared to pure aerobic exercise in children [68, 71] and adults [66]. The finding of the present longitudinal, but non-interventional study, that motor coordination was the best predictor for EF, supports the assumption of shared information processes in both motor and cognitive control [13] and the basic principles of the cognitive stimulation hypothesis [63–65]. Indeed, the results of a recent meta-analysis suggest that besides timed performance in movements and fine motor skills, bilateral body coordination is most strongly related to cognitive skills [29]. The authors explain their findings in terms of complex motor abilities, i.e. more information-oriented ones, having higher cognitive demands. More studies systematically varying the coordinative demands of specific physical activities and controlling for cognitive engagement [112, 113] are essential to conclusively answer the question of which of the two hypotheses are more probable [114].

The present study has limitations that need to be addressed. First, although the present study employed a longitudinal design, which is certainly superior to a cross-sectional design [72], not all variables were measured at every wave of data collection, which did not allow setting up an autoregressive mediation model. The strongest confounding variable in a mediational model is always the prior level of the dependent/exogenous variable. Future studies should include measures of all interesting variables at any wave of data collection and account for initial levels, e.g. of EF at t1 and academic achievement at t1 and t2, to reduce the potentially inflated estimates of the causal path of interest.

Second, inhibition and shifting were assessed by means of the same (child-adapted fish flanker) task–even if derived from two different blocks. Calculating two core EFs out of one single test is economical and more compatible with the testing time constraints imposed by teaching schedules at school. Nevertheless, this methodological decision might be the reason why the two measurement error terms showed high covariance.

Third, the same argument applies to the indicators used to assess children’s motor abilities: each single motor ability was measured by one single test. However, for the setup of the three separate models, several tests would have better reflected the width of the constructs of interest. Especially motor coordination is considered a broader construct than, for example, cardiovascular endurance. Further studies could tackle this problem by using not one, but all four tests of the KTK [80].

In conclusion, the present results that the relation of motor ability in general–and motor coordination in particular–to academic achievement is mediated by EF, add to the literature on the mechanisms underlying this relationship and highlights the centrality of motor coordination as early as childhood for positive trajectories of health development in multiple domains [50, 115]. Although the present study is non-interventional in nature, it is to consider that a high level of coordinative motor competence is the outcome of coordinatively challenging motor and sports training. Thus, the strong predictive validity of motor coordination for EF and of the latter for academic achievement suggests the usefulness of letting children engage in physical activities, which are both cognitively and coordinatively demanding and physically engaging.

## Supporting information

S1 Dataset Dataset underlying the findings of the current study. (SAV)
